# OCT on a chip aims at high-quality retinal imaging

**DOI:** 10.1038/s41377-021-00467-z

**Published:** 2021-01-21

**Authors:** Dierck Hillmann

**Affiliations:** 1 Institute of Biomedical Optics, University of Lübeck, Peter-Monnik-Weg 4, 23562 Lübeck, Germany; 2 Thorlabs GmbH, Maria-Goeppert-Straße 9, 23562 Lübeck, Germany

**Keywords:** Imaging and sensing, Integrated optics, Biophotonics

## Abstract

Optical coherence tomography (OCT) has become one of the most important techniques in ophthalmic diagnostics, as it is the only way to three-dimensionally visualize morphological changes in the layered structure of the retina at a high resolution. In addition, OCT is applied for countless medical and technical purposes. Recent developments pave the way for small-footprint OCT systems at significantly reduced costs, thereby extending possible use cases. Now, it appears increasingly likely that, in the near future, OCT will find its way into many more industrial and medical applications, including disease monitoring at home.

Currently, ophthalmologic clinics and doctor’s offices have to pay up to 100,000 USD to purchase a state-of-the-art optical coherence tomography (OCT) system for diagnosis and therapy monitoring of retinal and other ophthalmologic diseases. Reducing this price tag would open up exciting new opportunities and applications outside of the examination room^1^. For example, age-related macular degeneration (AMD) tends to progress unpredictably. Daily retinal monitoring by OCT at home might not only allow optimal treatment but also provide crucial insights into disease progression and the effects of medical interventions. Additionally, low-cost OCT could not only find its way into telemedicine but also enable widespread use of OCT in third-world countries or play an important role in large-scale production monitoring.

Most of the work in the last decades focused on improving the performance of OCT and was not aimed at reducing the cost and size of the OCT technique. Nevertheless, some improvements^2^ found their way into commercial devices, but OCT systems are still not available for <10,000 USD. Apart from using nonstandard techniques such as multireference OCT^3^ or full-field time-domain OCT^4,5^, changing components for cheaper alternatives or using more cost-effective production processes for standard spectrometer-based OCT (spectral-domain, SD-OCT) appears to be the most promising way to maintain high image quality while still producing systems in large numbers at reduced production costs.

Probably, the most promising approach to cut costs and size is to replace optical components of SD-OCT systems with photonic integrated circuits (PICs). This technique is comparable to the microchip production process but uses optical waveguide-based components instead of electrical circuits and transistors. In a sufficiently large number, multiple optical components can be fully integrated and produced at very low costs. However, to obtain a state-of-the-art SD-OCT system, several critical components are required, including a light source (superluminescent diode, SLD), an interferometer, scanners, and a spectrometer comprising grating, optics, and suitable photodiodes or a camera chip.

One of the most critical of these SD-OCT components is the spectrometer. It is the heart of any SD-OCT system and determines its performance. When implementing an OCT spectrometer with PICs, it is commonly realized as an arrayed waveguide (AWG)^6^.

While there has been progress in using AWGs in OCT since its first demonstration^7^, until now, SD-OCT imaging utilizing AWGs has fallen short of the magical number of ~90 dB for the sensitivity at an acquisition speed of more than 10 kHz, which is required for retinal imaging. Particularly in the wavelength regime suitable for this and when utilizing low-cost silicon-based camera sensors, AWG-based OCT thus far has barely reached an 80 dB sensitivity.

A recent paper by Elisabet Rank et al. ^8^ demonstrated a considerable step towards high-quality low-cost OCT on a chip. For the first time, using an integrated optical circuit based on silicon nitride in an AWG-based spectrometer, the research team surpassed the 90 dB barrier at a 34 kHz A-scan rate. They demonstrated high-quality retinal OCT as well as OCT angiography at a central wavelength of ~800 nm. The authors successfully implemented and tested two different spectrometer layouts on the chip. Additionally, the entire production process is CMOS compatible, allowing fully integrated production of most optical and electronic OCT components on a single chip in the future.

This is a big step forward on the still long road ahead for researchers to obtain a complete OCT system on a chip. Although probably being the most performance-critical component for the endeavor, a full integration needs to introduce a light source, a line-scan camera or a collection of photodiodes, and an interferometer including a reference arm on the chip. Outside of the integrated OCT chip itself, compact and inexpensive optics and a scanner may be needed, depending on the application. Nevertheless, with the recent development by Rank et al., it appears likely that these components can be brought together to realize a high-quality, low-cost OCT-on-a-chip device.

## References

[1] Chopra, R., Wagner, S. K. & Keane, P. A. Optical coherence tomography in the 2020s—outside the eye clinic. *Eye*10.1038/s41433-020-01263-6 (2020). 10.1038/s41433-020-01263-6 PMC7853067 33168975

[2] Kim S (2018). Design and implementation of a low-cost, portable OCT system. Biomed. Opt. Express.

[3] Neuhaus K (2017). Simultaneous *en-face* imaging of multiple layers with multiple reference optical coherence tomography. J. Biomed. Opt..

[4] Sudkamp H (2016). *In-vivo* retinal imaging with off-axis full-field time-domain optical coherence tomography. Opt. Lett..

[5] Vabre L, Dubois A, Boccara AC (2002). Thermal-light full-field optical coherence tomography. Opt. Lett..

[6] Smit MK (1988). New focusing and dispersive planar component based on an optical phased array. Electron. Lett..

[7] Nguyen VD (2011). Spectral domain optical coherence tomography imaging with an integrated optics spectrometer. Opt. Lett..

[8] Rank EA (2021). Toward optical coherence tomography on a chip: in vivo three-dimensional human retinal imaging using photonic integrated circuit-based arrayed waveguide gratings. Light. Sci. Appl.

